# Performance of TPP-Crosslinked Nano-chitosan for Simultaneous Nutrient Adsorption in Nutrient-Rich Wastewater

**DOI:** 10.1021/acsomega.6c04170

**Published:** 2026-09-15

**Authors:** Li Kai Chin, Mamoru Oshiki, Norli Ismail

**Affiliations:** † Environmental Technology Division, School of Industrial Technology, 377488 Universiti Sains Malaysia, Penang 11800, Gelugor, Malaysia; ‡ Division of Environmental Engineering, Faculty of Engineering, Hokkaido University, North-13, West-8, Kita-ku, Sapporo, 060-8628, Japan

## Abstract

Excessive dissolved nitrogen and phosphorus drive aquatic eutrophication, yet conventional treatments such as biological nitrification–denitrification and chemical precipitation are costly, generate secondary waste, and are not well suited to simultaneous multi-nutrient removal in a single step. Despite growing interest in chitosan-based adsorbents, the competitive adsorption behavior of ammonium nitrogen (NH_4_^+^–N), nitrate nitrogen (NO_3_^–^–N), and phosphate (PO_4_–P) coexisting in a single matrix has not been systematically evaluated under optimized conditions. This study synthesized nano-chitosan crosslinked with tripolyphosphate (NCS) via ionotropic gelation and evaluated its simultaneous removal of these nutrients from synthetic wet-market wastewater. Physicochemical characterization (FTIR, Transmission Electron Microscopy, Scanning Electron Microscopy, Dynamic Light Scattering, and zeta potential) confirmed stable nanoscale particles with surface properties favorable for ionic interaction. Adsorbent dose (0.05–0.10 g), nutrient concentration (30–90 mg L^–1^), and contact time (1–3 h) were optimized using Response Surface Methodology; all reduced models were significant (*p* < 0.001, *R* ^2^ = 0.950–0.973). Kinetic analysis showed NH_4_^+^–N was best described empirically by the PSO model, while NO_3_^–^–N uptake was slower. Single-solute modified Langmuir *q*_max_ values were 50.4, 90.2, and 48.5 mg g^–1^ for NH_4_^+^–N, NO_3_^–^–N, and PO_4_–P, respectively; capacities decreased substantially under multi-nutrient conditions due to competitive adsorption, underscoring the need to evaluate adsorbents under both single and multi-solute conditions for realistic performance assessment.

## Graphical abstract

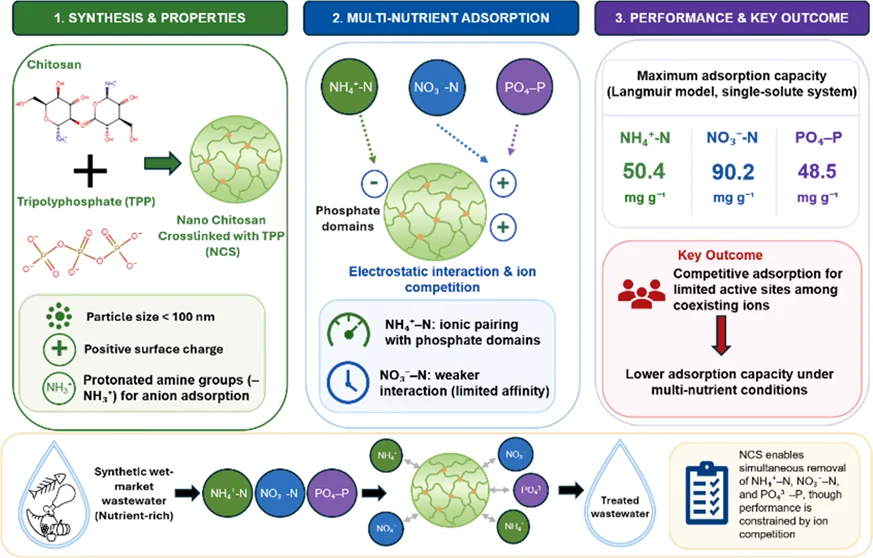

## 1 Introduction

Aquatic eutrophication is mainly the result of having too much dissolved inorganic nitrogen and phosphorus. This excess nitrogen and phosphorus results in shifts in the phytoplankton community and the rapid expansion of algal blooms in areas with high nutrient levels.^1,2^ The concentrations of ammonium, nitrate, and phosphorus in river or estuary systems are regularly monitored because they react differently in biogeochemistry.^2,3^ There is an increasing amount of research on different nutrient removal options, including adsorption, because of its operational ease, effectiveness in removing contaminants, and flexibility as an additional treatment method. In this context, chitosan and its nanostructured derivatives have gained considerable interest as biosorbents owing to their high density of surface functional groups and tunable ionic binding properties through formulation and crosslinking modifications.^4,5^ When adsorbing contaminants, chitosan nanoparticles will generally have a net positive surface charge, which is often used for drug delivery but can also be leveraged to electrostatically attract and bind to negatively charged contaminants.^4^ In addition to its increased surface area and shorter diffusion distances compared to bulk materials, nano-chitosan (NCS) has attracted growing interest due to its potential for adsorption applications. However, producing stable nano-chitosan requires careful control of colloidal formation and aggregation, which is commonly achieved using multivalent ions (e.g., sodium tripolyphosphate; TPP) in an ionotropic gelation process.

The most used ionic crosslinker for chitosan nanoparticles is TPP, and TPP-driven electrostatic attraction is the primary driving force for nanoparticle formation from chitosan.^6^ Experimental studies on ionotropic gelation have demonstrated that the TPP-to-chitosan ratio influences the size and properties of the resulting nanoparticle adsorbents, suggesting that the nanomaterial’s performance for pollutant adsorption can be optimized by adjusting the TPP-to-chitosan ratio. Recent research has indicated that if TPP is present in excess during nanoparticle fabrication, colloidal aggregation may occur and particle size distributions may change, potentially negatively impacting the ability of particles to adsorb pollutants.^7^ Stability testing also showed that NCS are dynamic colloids that may experience changes in physical and chemical characteristics over time and under varying temperature conditions, affecting adsorption performance.^6^ Although there is significant potential for TPP-crosslinked nano-chitosan to be a tenable adsorption platform, because of nutrient-specific adsorption behavior, ammonium (NH_4_^+^–N), nitrate (NO_3_^–^–N), and phosphate (PO_4_–P) differ significantly in charge, hydration, speciation, and the manner in which they interact with surface functional groups, one type of adsorbent may not have the same interaction with all forms of nutrients. In general, numerous studies have investigated the use of NCS as an adsorbent for nutrient removal from wastewater; however, most available studies have been conducted under single-solute (NO_3_^–^ or PO_4_) conditions, focusing on the removal of individual nutrient species in isolation.^8,9^ As a result, the competitive and interactive adsorption behavior of NH_4_^+^–N, NO_3_^–^–N, and PO_4_–P in multi-nutrient systems remains poorly understood. To date, there is limited research that systematically quantifies and compares the adsorption behavior of NH_4_^+^–N, NO_3_^–^–N, and PO_4_–P on NCS within a single wastewater matrix under controlled conditions while integrating statistical optimization, adsorption kinetics, and equilibrium isotherm analysis.

Consequently, the present study aims to evaluate the feasibility of applying NCS to simultaneously remove NH_4_^+^–N, NO_3_^–^–N, and PO_4_–P from synthetic wastewater, designed to better represent realistic multi-nutrient environments rather than idealized single-pollutant systems. The relatively nitrogen- and phosphorus-rich concentrations (30–90 mg L^–1^) were selected to reflect the concentration ranges commonly found in wet-market wastewater and to enable systematic evaluation of adsorption behavior under multi-nutrient conditions. The current study includes the synthesis and characterization via ionotropic gelation of NCS and the evaluation of its adsorption performance for NH_4_^+^–N, NO_3_^–^–N, and PO_4_–P from synthetic wastewater through the use of Response Surface Methodology (RSM), adsorption kinetics, and equilibrium isotherm analyses; this research will generate valuable knowledge about the performance of NCS for use in tertiary nutrient polishing applications.

## 2 Materials and Methods

### 2.1 Materials

Chitosan (molecular mass 10–20 kDa; DDA≥90%) was purchased from Bio Basic, Canada. Sodium tripolyphosphate (TPP) was purchased from Evergreen Engineering & Resources, Malaysia.

### 2.2 Preparation of NCS

In brief, the ionotropic gelation method was used to prepare the NCS as previously described.^10^ This ionotropic gelation method has been utilized to produce the NCS because it is simpler and less toxic to the environment.^6,11–13^ The overall preparation of NCS followed the studies from Bouillons-Gamboa et al. (2024) and Koukaras et al. (2012) with some modifications. The ratio between CS and TPP was set to 3:1 (w/w). Briefly, 0.25 g of chitosan was dissolved in 100 mL of 1% (v/v) acetic acid and stirred overnight to obtain a clear solution. The solution was subsequently filtered through a 0.45 μm membrane to remove undissolved chitosan. The pH of the chitosan stock solution was raised from approximately pH 2.5 in 1% v/v acetic acid to 4.0–4.5 by the dropwise addition of dilute sodium hydroxide to enhance the crosslinking efficiency and facilitate the formation of smaller NCS particles. Several studies have reported that conducting the crosslinking reaction under acidic conditions yields smaller, more stable NCS, due to improved protonation of amino groups and enhanced interaction with crosslinking agents.^11,14,15^ Separately, 83.3 mg of TPP was dissolved in 10 mL of distilled water. The TPP solution was added dropwise to the chitosan solution under continuous stirring at 1000 rpm for 45 min at 25 °C. The resulting suspension was centrifuged at 14,000 rpm for 15 min to collect the NCS particles. The NCS was washed twice with distilled water and stored in a fridge at 4 °C.

### 2.3 Characteristics of the Samples

Several analyses were performed to compare the hydrodynamic size distribution, core size and shape, zeta potential, and chemical composition across three sample types: CS (unmodified bulk chitosan), NCS (TPP-crosslinked nano-chitosan), and NCS-A (TPP-crosslinked nano-chitosan after nutrient adsorption). Dynamic Light Scattering (DLS) (Malvern Zetasizer, UK) was used to measure hydrodynamic particle size, a technique that measures light scattering from the Brownian motion of particles in a liquid suspension. This is important for evaluating the uniformity and quality of dispersion of the synthesized nanoparticles. The zeta potential was evaluated using the Malvern Zetasizer (UK) to assess the surface charge and colloidal stability of the NCS. High absolute zeta potential values indicate better particle stability, thus decreasing the likelihood of aggregation. Dispersions with |ζ| ≥ 30 mV are generally considered electrostatically stable due to sufficient interparticle repulsion.^16^ To provide information about the core size and shape of the NCS, a Transmission Electron Microscope (TEM) JEM-F200 (Japan Electron Optics Laboratory) was used for high-resolution imaging in order to view the actual size, shape, and structure of the nanoparticles. Fourier Transform Infrared (FTIR) – Bruker VERTEX 70 was also used to identify functional groups and confirm the chemical composition of the NCS. In addition to this, Scanning Electron Microscopy (SEM) – ZEISS EVO 15 was performed to assess the surface morphology and structure of the CS at a microscopic level.

### 2.4 Experimental Setup for Adsorption

Batch adsorption experiments were performed using 250 mL of synthetic wastewater in each test, with continuous magnetic stirring at 250 rpm and 25 °C. The adsorption suspension was maintained under mildly acidic conditions, with the initial pH adjusted to approximately 4.0–5.0 before contact with NCS. Synthetic wastewater was prepared by dissolving ammonium chloride (NH_4_Cl), sodium nitrate (NaNO_3_), and potassium dihydrogen phosphate (KH_2_PO_4_) in deionized water to achieve initial concentrations of 30–90 mg L^–1^ for each nutrient. NCS dosage was based on dry mass: a known volume of NCS suspension was dried to constant weight to determine solids concentration (g L^–1^), and the appropriate suspension volume was then added to provide 0.05–0.10 g of dry NCS per experiment. For each batch test, 250 mL of wastewater was contacted with NCS for 1–3 h, after which solids were separated by centrifugation at 14,000 rpm for 15 min. Dissolved NH_4_^+^–N, NO_3_^–^–N, and PO_4_–P were measured using a HACH DR900 Colorimeter, including calibration procedures and detection limits. Adsorption capacity was calculated using eq 1:

1 $$q_{e}=\frac{\left(C_{o}-C_{e}\right)\cdot V}{m}$$

*q*_e_ represents adsorption capacity (mg of adsorbate per g of adsorbent), *C*_0_ represents the concentration of the adsorbate in the solution (mg L^–1^), *C*_e_ represents the equilibrium concentration of the adsorbate in the solution (mg L^–1^), *V* represents the volume of the solution (L), and *m* represents the mass of the adsorbent (g).^17^

### 2.5 RSM

The Box–Behnken Design (BBD) was employed using three numerical factors: Initial dose in the range of 0.05–0.1 g, contact time between NCS and wastewater ranging between 1 and 3 h, and initial pollutant concentration ranging from 30 to 90 mg L^–1^. The BBD comprised 17 experimental runs in total, including five center-point runs consisting of one center-point run and four additional replicates (Runs 2, 5, 6, 7, and 16). These center-point replicates are a standard feature of the BBD and were included to provide an independent estimate of pure experimental error and to assess whether significant model curvature was present within the design space.^18^

### 2.6 Optimization

The adsorption experiment was conducted under the optimized conditions obtained from the RSM model. The following eq 2 was used to calculate the percentage difference between the predicted and experimental results:

2 $$Percentage\;differences\;\left(\%\right)=\left|\frac{Y_{\exp}-Y_{pred}}{Y_{pred}}\right|\times 100$$

### 2.7 Adsorption Kinetics

Kinetics behavior was evaluated using the pseudo-first-order (PFO) and pseudo-second-order (PSO) models, which are commonly applied to describe adsorption rate characteristics.^19,20^ Both PFO and PSO model parameters were determined by nonlinear regression, applied directly to the nonlinear forms of the equations (eqs 3 and 4). This approach was adopted to avoid the bias and error introduced by the traditional linear transformations of these models. The PFO assumes that the adsorption rate is directly proportional to the difference between the equilibrium adsorption capacity and the amount adsorbed at time (t).^21^ The nonlinear form of the equation is expressed as eq 3:

3 $$q_{t}=q_{e}\left(1-e^{-k_{1}t}\right)$$

where *q*_*e*_ (mg g^–1^) is the equilibrium adsorption capacity, *q*_t_ (mg g^–1^) is the adsorption capacity at time *t*, and *k*_1_ (min^–1^) is the PFO rate constant. The model parameters (*q*_e_ and *k*_1_) were obtained by fitting the experimental data to the nonlinear expression using the least-squares method, and the goodness of fit was evaluated using the coefficient of determination (*R* ^2^) and the root-mean-square error (RMSE).

Although originally proposed as an empirical model, the pseudo-second-order (PSO) model has been theoretically rationalized for sorption systems under specific conditions, as demonstrated by Azizian et al. (2004). Therefore, fitting adsorption data to the PSO model should not be interpreted as direct evidence of chemisorption. Rather, the PSO model describes the adsorption rate behavior, and its parameters may reflect combined effects of adsorption, desorption, and initial solute concentration.^20,22^ Its nonlinear form is expressed in eq 4.

4 $$q_{t}=\frac{q_{e}^{2}k_{2}t}{1+q_{e}k_{2}t}$$

where *k*_2_ (g mg^–1^ min^–1^) is the PSO rate constant and *q*_e_ is the equilibrium adsorption capacity. The parameters *q*_e_ and *k*_2_ were obtained through nonlinear regression analysis by minimizing the residual sum of squares between experimental and predicted *q*_*t*_ values. The model that exhibited the highest *R* ^2^ value and the lowest RMSE was considered to best describe the adsorption kinetics.

### 2.8 Adsorption Isotherms

The adsorption isotherm study was conducted to evaluate the equilibrium adsorption behavior of NH_4_^+^–N, NO_3_^–^–N, and PO_4_–P onto NCS under the optimized experimental conditions. The Langmuir framework assumes that adsorption proceeds as a monolayer on an energetically homogeneous surface containing a finite number of identical active sites, with each site accommodating a single adsorbate molecule and no lateral interactions occurring between adsorbed species. Although the classical Langmuir isotherm has been widely applied to liquid-phase adsorption data, it was originally derived under gas-phase assumptions, and its direct application to liquid–solid systems has been shown to introduce thermodynamic inconsistencies.^23^ In particular, the classical model assumes that the desorption rate depends solely on surface coverage and does not account for the influence of bulk adsorbate concentration, which is physically inconsistent with liquid-phase systems. The modified Langmuir isotherm proposed by Azizian et al. (2018) addresses this by revising the desorption rate to be proportional to *C*_s_ – *C*_e_, thereby incorporating the driving force of bulk concentration on the desorption process. While retaining the fundamental monolayer assumptions described above, this modification also normalizes the equilibrium concentration against the saturation concentration of the adsorbate, yielding a dimensionless affinity constant consistent with IUPAC requirements for thermodynamic calculations. The nonlinear regression form of the modified Langmuir isotherm used for parameter estimation is expressed as eq 5:

5 $$q_{e}=\frac{q_{\max}K_{ML}C_{e}}{C_{s}+\left(K_{ML}-1\right)C_{e}}$$

where *q*_e_ (mg g^–1^) is the equilibrium adsorption capacity, *q*_max_ (mg g^–1^) is the theoretical maximum adsorption capacity, *K*_ML_ is the dimensionless modified Langmuir affinity constant, *C*_e_ (mg L^–1^) is the equilibrium concentration of the adsorbate in solution, and *C*_s_ (mg L^–1^) is the saturation concentration of the adsorbate in the liquid phase.

To describe how nutrients adsorb to NCSs in equilibrium, the Freundlich isotherm model was used. The Freundlich isotherm model does not assume either a homogeneous surface or a monolayer of the adsorbed material like the Langmuir model; instead, the Freundlich model assumes that adsorption occurs on a nonhomogeneous surface of the NCS. As an empirical model, the Freundlich isotherm further assumes that the higher-energy binding sites are occupied preferentially and that the effective adsorption energy decreases logarithmically with increasing surface coverage; accordingly, it does not impose a finite monolayer capacity on the adsorbent. This model is particularly useful when the adsorbent has many different types of surface functional groups that affect the adsorption process.^24^ Equation 6 presents the Freundlich equation, which relates the equilibrium adsorption capacity of the sorbent material (*q*_e_, expressed as mg of pollutant adsorbed per g of sorbent) to the equilibrium concentration of the pollutant in solution (*C*_e_). The model includes the Freundlich constant (*K*_F_), which is indicative of the adsorption capacity, and the heterogeneity factor (1/*n*), which reflects the adsorption intensity and surface heterogeneity. Values of 1/*n* less than unity indicate favorable adsorption behavior as stated in eq 6.

6 $$q_{e}=K_{F}C_{e}^{1/n}$$

### 2.9 Competitive Adsorption Suppression Index

To evaluate the influence of ion coexistence on adsorption performance, a suppression index was calculated as in eq 7.

7 $$Comparison\;suppression=1-\frac{q_{multi}}{q_{single}}$$

where *q*_single_ represents the adsorption capacity obtained under single-solute conditions (isotherm experiments) and *q*_multi_ represents the adsorption capacity measured under simultaneous multi-nutrient conditions at the optimized operating parameters.^25^

## 3 Results and Discussion

### 3.1 Material Characteristics of CS, NCS, and NCS-A

The physicochemical characteristics of CS, NCS, and NCS-A were investigated to confirm successful nanoparticle formation and ionic crosslinking, as well as to elucidate properties relevant to nutrient adsorption.

#### 3.1.1 Fourier Transform Infrared (FTIR) Spectroscopy

FTIR spectra of CS, NCS, and NCS-A were recorded over the range 400–4000 cm^–1^ as shown in Figure 1. Native chitosan exhibited the characteristic polysaccharide fingerprint reported in the literature, including a broad O–H/N–H stretching band in the 3200–3500 cm^–1^ region, weak C–H stretching near 2920–2870 cm^–1^, amide I (C–O) and amide II (N–H bending) bands around 1650 and 1590 cm^–1^, CH_2_/CH_3_ bending near 1470–1390 cm^–1^, and strong saccharide C–O–C/C–O vibrations between 1200 and 950 cm^–1^.^26,27^

**Figure 1:**
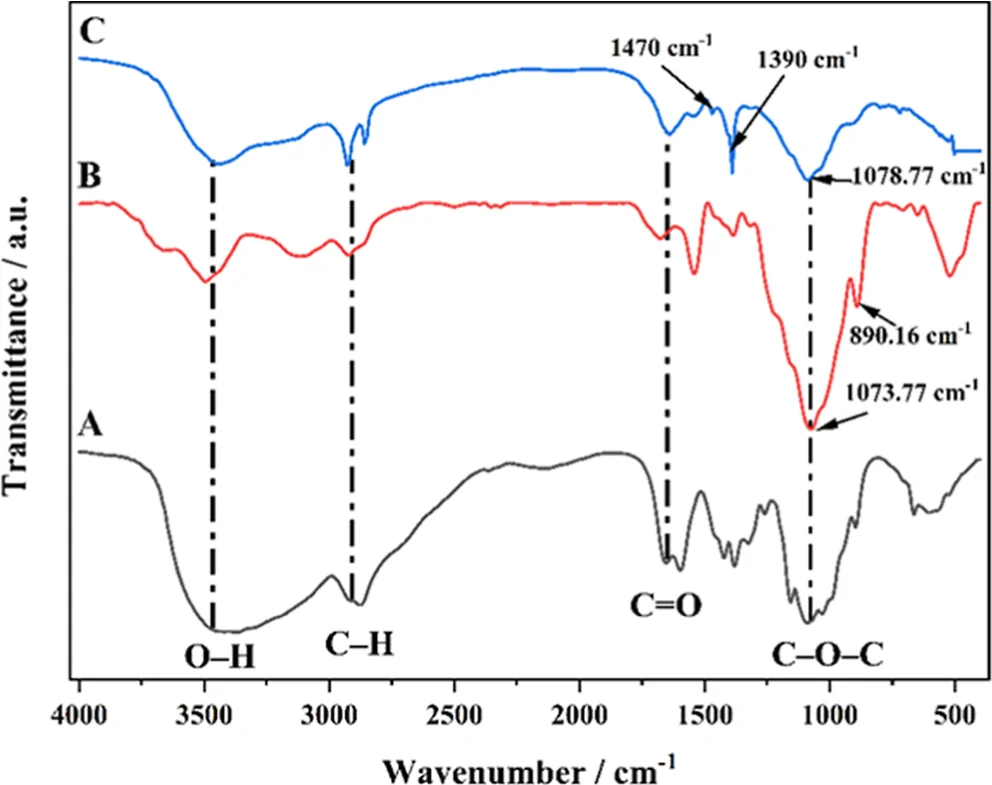
FTIR spectra (A) CS, (B) NCS, and (C) NCS-A.

Upon ionic crosslinking with TPP to form NCS, diagnostic spectral changes confirmed the formation of the chitosan–TPP polyelectrolyte complex. These included shifts in the amide I and amide II/N–H bending bands, broadening and red-shifting of the O–H/N–H stretching envelope, and the appearance of phosphate-related bands in the 1100–900 cm^–1^ region, consistent with electrostatic interactions between protonated chitosan amines and polyphosphate groups.^28,29^

After adsorption (NCS-A), additional spectral changes were observed, including further broadening of the O–H/N–H stretching region and changes in bands near 1470 and 1390 cm^–1^, which are consistent with interactions between the NCS surface and adsorbed inorganic nitrogen species.^30^ In the phosphate region, a slight shift of the P–O stretching band and attenuation of the P–O–P feature suggests involvement of phosphate groups in adsorption interactions.^31^ The shift in the N–H bending band (∼1590 cm^–1^) and attenuation of the P–O–P feature in NCS-A provide supporting spectral evidence that protonated amino groups (−NH_3_^+^) serve as primary binding sites for anionic NO_3_^–^, H_2_PO_4_^–^, and HPO_4_^2–^, while the negatively charged phosphate domains introduced through TPP crosslinking facilitate ionic pairing with NH_4_^+^ cations. Overall, the observed spectral changes before and after adsorption support successful TPP crosslinking and subsequent nutrient uptake by nano-chitosan through surface-mediated interactions, such as electrostatic attraction and hydrogen bonding.

Figure 2 summarizes the proposed dual-site adsorption mechanism of NCS. The chitosan backbone provides protonated –NH_3_^+^ groups that electrostatically attract anionic NO_3_^–^–N and PO_4_–P, while phosphate domains introduced by TPP crosslinking provide negatively charged sites that retain NH_4_^+^–N through ionic pairing. This dual-site mechanism allows a single nano-adsorbent to simultaneously capture both cationic and anionic nutrients.

**Figure 2:**
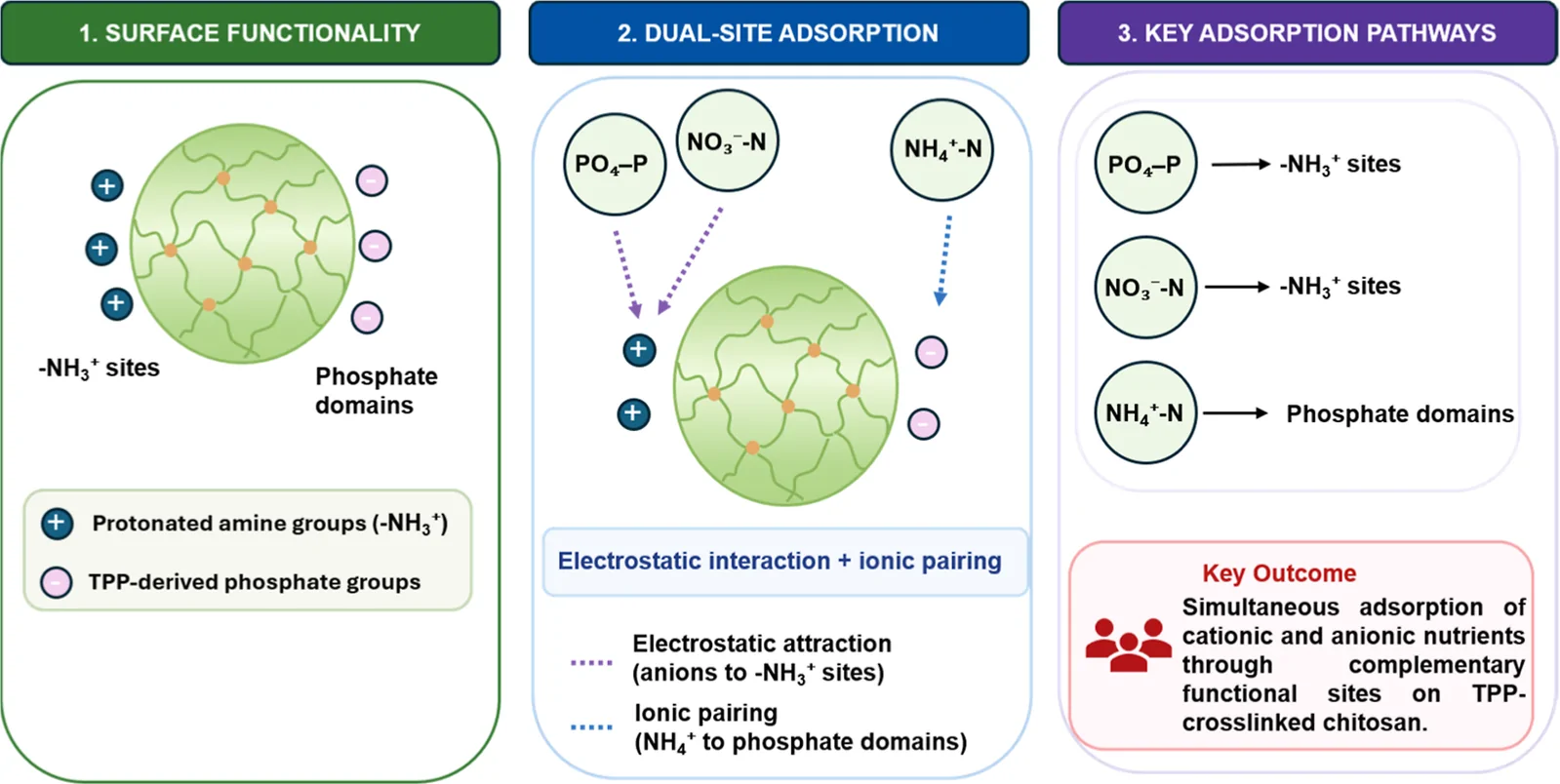
Proposed adsorption mechanism of NCS: (1) surface functional groups, (2) dual-site binding of cationic and anionic nutrients, and (3) summary of adsorption pathways.

#### 3.1.2 Morphology Analysis

SEM and TEM were utilized to analyze the surface structure of CS, NCS, and NCS-A. Images of the different sample types are shown in Figure 3a–f, and particle diameter measurements were completed using ImageJ by applying the scale bars from the TEM images. Bulk native chitosan is depicted in Figure 3a,b via SEM images. The large, irregularly shaped particles are rough and heterogeneous, characteristics commonly observed for native CS.

**Figure 3:**
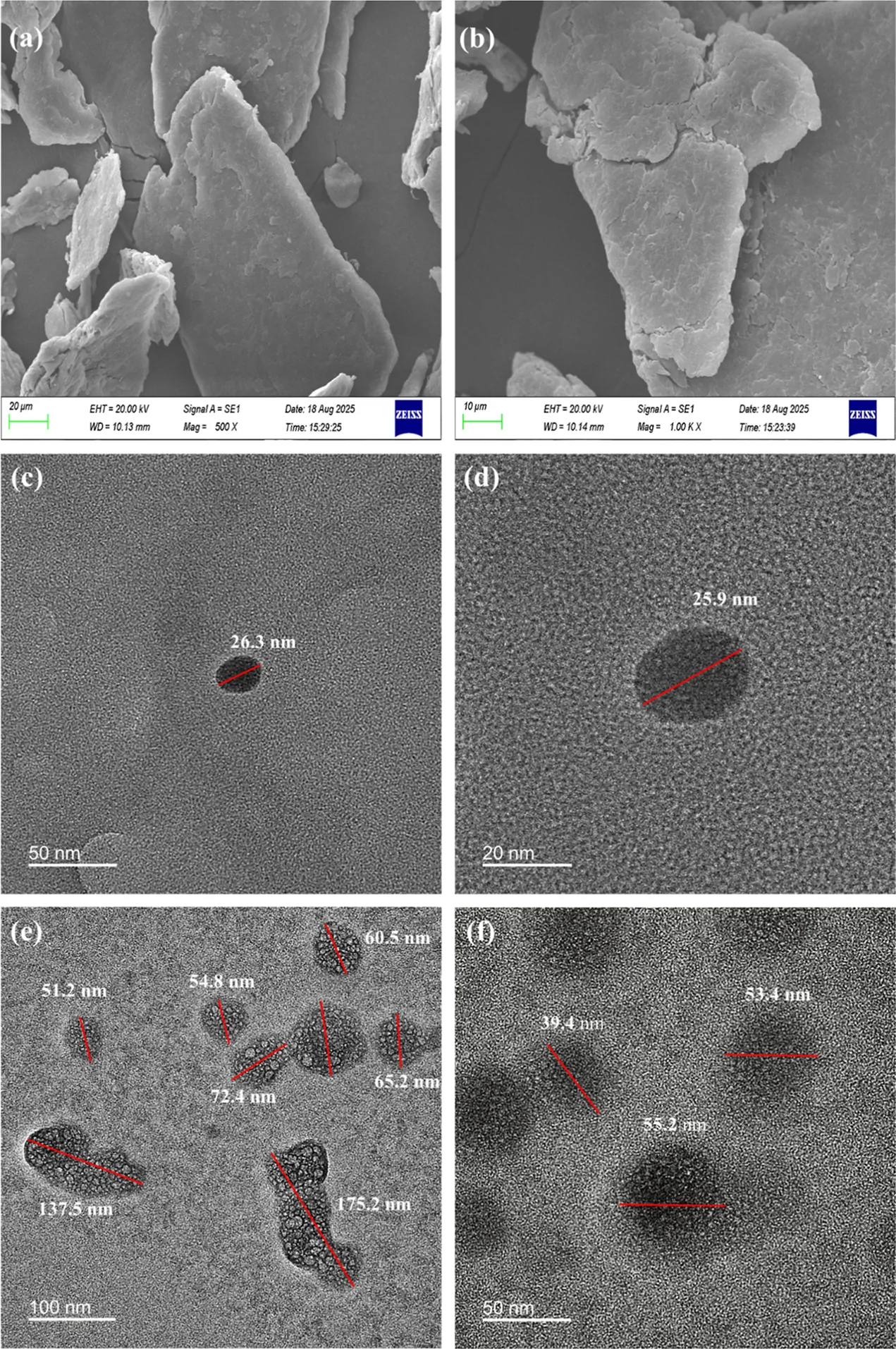
(a,b) SEM of CS, (c,d) TEM of NCS, and (e,f) TEM NCS-A.

Conversely, NCS images (Figure 3c,d) taken with the TEM show well-defined, approximately spherical nanoparticles in the nanometer size range, with smoother surfaces and therefore improved dispersion. These findings are consistent with previous studies, whereby incorporating TPP via ionotropic gelation produced stable chitosan nanospheres with a greater surface area available for interaction.^10,32^ These benefits related to nanoscale particle characteristics provide more available adsorption sites and shorter diffusion distances.^33^ Adsorption of nutrients has produced aggregate nanoparticles (Figure 3e,f), as well as a relatively small increase in the apparent nanosphere size. This aggregation may be associated with partial neutralization of the nanoparticle surface charge following nutrient adsorption, which would reduce electrostatic repulsion and promote particle–particle association.

Similar aggregation behavior has been reported for NCS-TPP systems upon addition of charged ions and has been attributed to electrostatic interactions and ion bridging effects.^34^ Although the aggregated NCS-A particles retained visually identifiable spherical features, the TEM images alone cannot confirm that the TPP-crosslinked network remained structurally intact or exclude material degradation during adsorption. Previous studies have reported that TPP-crosslinked NCS nanoparticles can maintain particle integrity under certain adsorption conditions.^35^ However, the applicability of these findings to the present material should be interpreted cautiously because mechanical stability and structural degradation were not directly evaluated in this study. The observation of bright regions of interest in the nanoparticle images following nutrient adsorption results from localized variations in electron density caused by polymer swelling and the formation of an inhomogeneous distribution of ions adsorbed onto the NCS matrix. The region of brightness is consistent with previous studies on TEM images of polymeric nanoparticles, where adsorbed ions interact with the polymer or the structure reorganizes, thus producing local differences in electron density and thickness, and hence resulting in either bright or dark regions within the TEM images.^36^ Overall, the transition from relatively discrete NCS particles to aggregated nutrient-loaded NCS-A indicates changes in particle association and apparent morphology. These observations support the occurrence of surface-mediated interactions but do not independently confirm the adsorption mechanism, structural integrity, or reversibility of the observed aggregation.

#### 3.1.3 Hydrodynamic Particle Size and Zeta Potential

Figure 4 shows the hydrodynamic particle size and zeta potential for the samples. CS exhibited an average hydrodynamic diameter of approximately 224.5 nm and a positive zeta potential of 11.43 mV, suggesting limited electrostatic stabilization and thus a tendency toward aggregation in aqueous suspension.^37^ The moderate surface charge of CS provides insufficient electrostatic repulsion to maintain colloidal suspension stability, thereby promoting aggregation (Figure 4a). By comparison, NCS exhibited a significantly lower average hydrodynamic diameter of 107.49 nm (Figure 4b), consistent with the formation of discrete chitosan nanoparticles via ionic crosslinking/ionotropic gelation with polyanions such as sodium tripolyphosphate (TPP), which compacts and stabilizes chitosan chains into nanoscale particles. NCS also had a significantly higher zeta potential of 32.38 mV compared to CS. This improvement in colloidal stability may be attributed to TPP crosslinking, which induces a structural reorganization and increases the degree of uniformity of the nanoparticles, and provides effective exposure of protonated amino groups present on the surface of the nanoparticles.^38^ The positive surface charge of NCS (+32.38 mV) at the operational pH (∼4–5) has direct implications for the adsorption behavior of each nutrient species. At this pH range, NH_4_^+^ is the dominant ammonium species (p*K*a NH_4_^+^/NH_3_ = 9.25), H_2_PO_4_^–^ and HPO_4_^2–^ are the predominant phosphate forms, and NO_3_^–^ remains fully dissociated. The net positive NCS surface is therefore electrostatically favorable for the attraction of anionic NO_3_^–^ and phosphate species, whereas direct electrostatic adsorption of NH_4_^+^ onto the protonated chitosan surface (−NH_3_^+^) is unfavorable due to like-charge repulsion. NH_4_^+^ uptake is instead attributed to ionic pairing with the negatively charged phosphate domains introduced through TPP crosslinking. Together, these speciation conditions are consistent with the observed adsorption behavior and support the dual-site ionic interaction mechanism proposed for the simultaneous removal of NH_4_^+^–N, NO_3_^–^–N, and PO_4_–P by NCS. Besides that, the average size and zeta potential of the reported NCS by Des Bouillons-Gamboa et al. (2024) were also similar, i.e., 178 ± 18 nm and 45 ± 4 mV, respectively.

**Figure 4:**
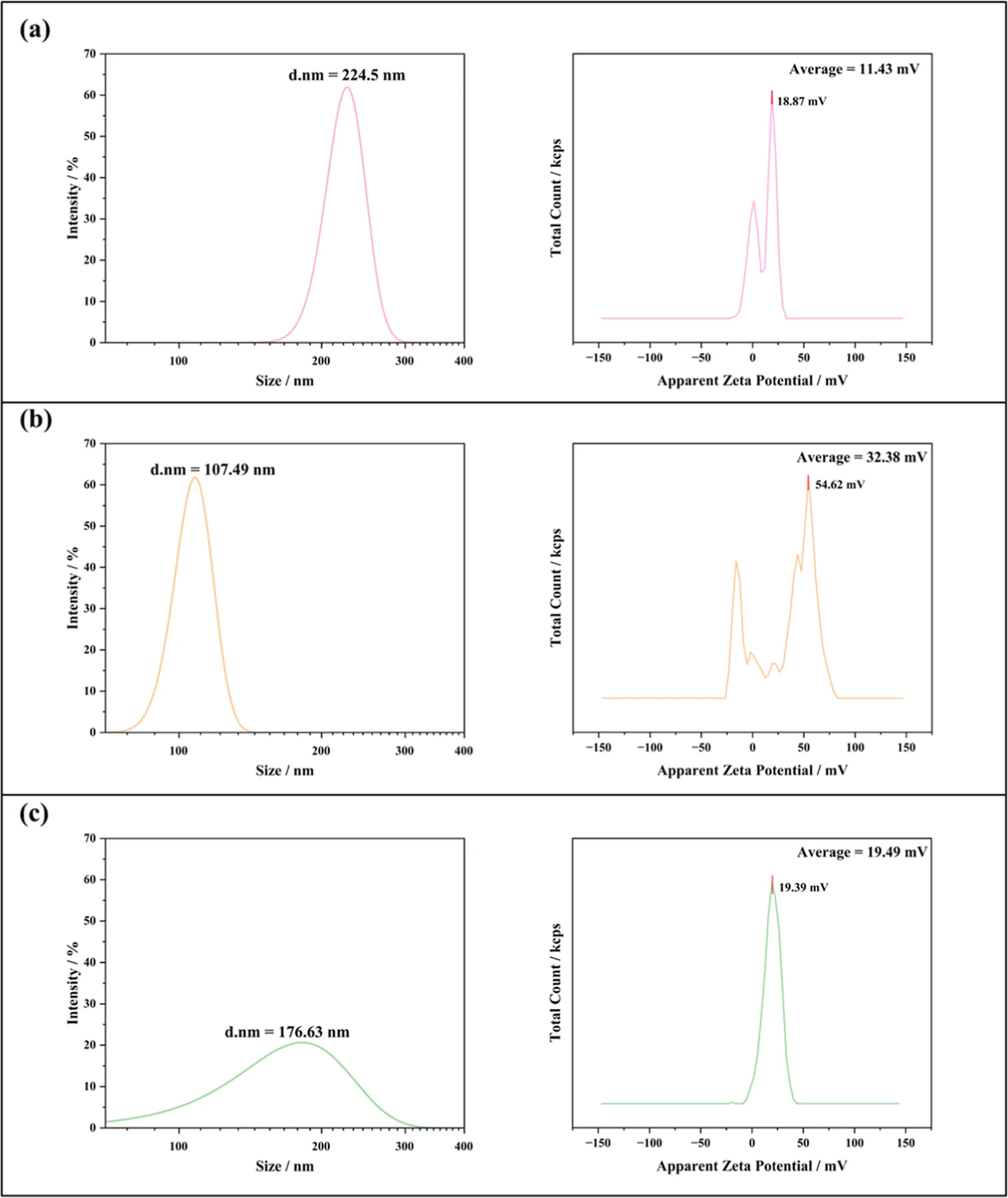
DLS size distribution and zeta analysis of (a) CS, (b) NCS, and (c) NCS-A.

Upon nutrient adsorption, there was an increase in average hydrodynamic diameter to 176.63 nm (Figure 4c) due to partial swelling and surface loading with the presence of the inorganic nutrient species. The increase in average hydrodynamic diameter was likely due to electrostatic interactions between protonated chitosan amino groups and adsorbed ions, combined with localized water entrapment within the polymer matrix.^34^ There was also a concomitant decrease in zeta potential to 19.49 mV, indicating the partial neutralization of surface charge as a result of the adsorption of anionic phosphate and nitrate species, combined with ionic interactions involving ammonium. A decrease in surface charge would reduce electrostatic repulsion between the nanoparticles, thereby promoting particle–particle association. However, despite particle aggregation, no obvious loss of particle morphology was observed under the conditions examined. This observation indicates that clear morphological collapse was not evident in the TEM images, but it does not confirm preservation of the TPP-crosslinked network or exclude material degradation. The observed aggregation therefore appears to be mainly associated with changes in interparticle interactions rather than obvious morphological collapse. Nevertheless, the reversibility of this aggregation and the regeneration potential of the material cannot be confirmed from the present results and should be evaluated through dedicated desorption and reuse experiments. Overall, the findings indicate effective nutrient uptake, although some reduction in colloidal stability occurred.

### 3.2 RSM

In this study, RSM was conducted to determine the optimal conditions for removing NO_3_^–^–N, NH_4_^+^–N, and PO_4_–P from synthetic wastewater using NCS. Table 1 presents the experimental design and the response yield. In general, the NO_3_^–^–N adsorption performance obtained in this study is within the range reported for moderately modified chitosan materials operating under mildly acidic to near-neutral conditions. For example, iron-oxide/chitosan bead systems have reported capacities on the order of ∼47 mg g^–1^ for nitrate at pH values below approximately 5.5,^39^ indicating that the present NCS exhibits comparable performance despite the absence of heavy metal loading or specialized ion-exchange functionality.

**Table 1:** Adsorption Capacity of NCS Based on the Experimental Design from RSM

Run	Factor 1	Factor 2	Factor 3	Response: Adsorption Capacity, mg g^–1^		
	A: Initial dose (g)	B: Initial concentration (mg L^–1^)	C: Contact time (hours)	NO_3_^–^–N	NH_4_^+^–N	PO_4_–P
1	0.10	30	2	29.6	6.57	2.08
2	0.075	60	2	26.5	9.78	4.64
3	0.05	30	2	24.8	3.41	1.34
4	0.075	30	1	17.0	4.15	1.48
5	0.075	60	2	26.8	8.68	4.26
6	0.075	60	2	26.3	8.38	4.18
7	0.075	60	2	25.3	7.79	4.32
8	0.05	60	3	28.6	8.12	4.12
9	0.075	30	3	30.5	5.39	2.13
10	0.10	60	3	38.4	12.4	5.12
11	0.075	90	1	26.0	7.24	3.24
12	0.075	90	3	35.1	8.71	4.64
13	0.05	60	1	24.1	6.22	4.25
14	0.05	90	2	28.4	7.82	3.98
15	0.10	90	2	32.5	11.3	4.86
16	0.075	60	2	24.8	7.92	4.21
17	0.10	60	1	19.5	9.78	2.97

For NH_4_^+^–N, the maximum observed capacity of 12.4 mg g^–1^ falls within the mid-range of values reported for chitosan composites incorporating mineral cation-exchange components. Literature reports for mordenite-chitosan composites typically are approximately 17.7 mg g^–1^, suggesting that the adsorption behavior of NCS is consistent with that of chitosan materials possessing moderate cation-exchange characteristics.^40^

In contrast, the PO_4_–P adsorption capacity observed in this work is lower than that reported for engineered chitosan sorbents specifically designed for phosphate capture. Such materials incorporate calcium, rare-earth, or metal-binding functional groups, for instance, CaCO_3_ containing chitosan composites, for which reported maximum capacities commonly reach several tens of mg g^–1^.^41^ The comparatively lower capacity observed here is therefore consistent with the absence of dedicated phosphate-binding sites in the present material.

The data gathered indicates that the performance of NCS adsorbent is as good as that of other types of adsorbents for NO_3_^–^–N and NH_4_^+^–N when compared with non-modified and modified chitosan-based adsorbents; however, with respect to the ability of NCS sorbent to remove PO_4_–P, it does not perform as good as engineered phosphate selective sorbents. It is worth noting that many of the literature values were obtained from experiments involving single nutrients, whereas this experiment evaluated multiple nutrients simultaneously. Thus, the values reported represent how the NCS adsorbs under conditions of competition for the same or similar sites versus what their true/site-based characteristics would be if tested individually.

To check how well the reduced quadratic polynomial models fit with the biosorbent experimental data for NO_3_^–^–N, NH_4_^+^–N, and PO_4_–P, an analysis of variance (ANOVA) was conducted. Hierarchical backward elimination was used to refine the regression models by removing non-significant (*p* > 0.05) higher-order terms not needed to demonstrate significant interaction or quadratic effects, while retaining the necessary lower-order terms to maintain model hierarchy. All three models were determined to be statistically significant, with their respective F-values (NO_3_^–^–N = 47.1; NH_4_^+^–N = 42.0; PO_4_–P = 45.2; *p* < 0.001), indicating that the selected independent variables, when combined, sufficiently account for the total variability in the adsorbent’s capacity to remove these contaminants from an aqueous environment. Initial pollutant concentration (B) and adsorbent dosage (A) also had significant effects on the total amount of nitrate adsorbed, as indicated by linear and quadratic regression models. Additionally, there were significant interaction effects between contact time and dosage for the adsorption of NO_3_^–^–N, indicating that the adsorption of this contaminant to the biosorbent is influenced by a time-dependent and dosage-dependent relationship. On the other hand, NH_4_^+^–N was most strongly affected by the amount of adsorbent (A), and the quadratic effect suggested that the dose–response relationship between the concentration of this contaminant and the amount of adsorbent used is nonlinear. The adsorption of PO_4_–P demonstrates a similar trend to NH_4_^+^–N; however, there was an even greater sensitivity of the amount of adsorbent to the initial concentration of contaminant (B), the amount of time (C), and their interaction (AC), indicating that phosphate uptake by the biosorbent is governed by both concentration-dependent and time-dependent processes. The validity of all models was further confirmed through high regression coefficients (*R* ^2^ = 0.950–0.973), a good agreement between adjusted and predicted *R* ^2^ values, and the measured precision value was greater than the required precision value of 4, all signs that the models developed can be used to successfully guide the experimentation conducted within this study. Full ANOVA results and fit statistics are provided in Table S1 (Supporting Information).

#### 3.2.1 Model Validation under Optimized Conditions

Experiments were performed to confirm the accuracy of the RSM model, using data from the design-expert numerical-optimization approach to identify the conditions expected to produce optimal experimental results. The three conditions selected were an adsorbent dosage of 0.1 g, a contact time of 2.98 h, and an initial pollutant concentration of 73.7 mg L^–1^. Each test was done in triplicate. The average value of all three tests per pollutant was used to compare the RSM model’s prediction with the experimental result, as explained in eq 2. As presented in Table 2, the measured adsorption capacity for each pollutant investigated likewise predicted by the model (all results were ≤10% difference). With respect to the experimental value for NO_3_^–^–N (37.8 mg g^–1^), this result was very close to the predicted value (39.3 mg g^–1^) and indicated an acceptable 3.82% difference, therefore supporting the model’s adequacy for prediction within the design space. Similarly, for NH_4_^+^–N, the measured value (11.5 mg g^–1^) was close to the predicted value (12.4 mg g^–1^), representing a 7.26% difference and falling within the acceptable range for RSM validation. The case of PO_4_–P was also very similar in that the measured value (5.23 mg g^–1^) had a good agreement with the predicted value (5.80 mg g^–1^) and a 9.83% difference. In conclusion, every pollutant that was measured to determine the relationship between predicted responses from the RSM model and experimental responses from the experimental tests had a percentage difference less than 10%, indicating that the RSM model is reliable and robust in terms of accurately predicting the adsorption performance under the specified optimum conditions for pollutant adsorbates.

**Table 2:** Validation of RSM-Predicted and Experimental Adsorption Capacities for Different Pollutants Under Optimized Conditions

Type of pollutant	Response, adsorption capacity (mg g^–1^)		Percentage differences (%)
	Predicted	Experimental	
NO_3_^–^–N	39.3	37.8	3.82
NH_4_^+^–N	12.4	11.5	7.26
PO_4_–P	5.80	5.23	9.83

### 3.3 Adsorption Kinetics

Tables 3 and 4 present the kinetics parameters obtained from the pseudo-first-order (PFO) and pseudo-second-order (PSO) models for nutrient adsorption onto NCS. Although both models yielded high coefficients of determination (*R* ^2^ ≈ 0.99), *R* ^2^ alone is an insufficient criterion for model validity; a physically sound model must also produce a predicted equilibrium capacity (*q*_e,cal_) that closely agrees with the experimentally measured value (*q*_e,exp_). The substantial discrepancies observed here are most likely attributable to the kinetics experiments not extending to a true adsorption plateau. The data capture only the initial rapid uptake phase and do not fully resolve the approach to genuine equilibrium, causing both models to overestimate *q*_e,cal_ through extrapolation beyond the measured time range.

**Table 3:** Kinetic Model Parameters for Nutrient Adsorption on the NCS^a^

Model	Response	Parameters				
		***q***_**e**,**exp**_ (mg g^–1^)	***q***_**e**,cal_ (mg g^–1^)	*k*_1_ (min^–1^)	*R* ^2^	RMSE
pseudo-1st order kinetic	NH_4_^+^–N	31.4	34.0	0.0137	0.99899	1.01
	NO_3_^–^–N	45.5	64.1	0.00537	0.99849	1.84
	PO_4_–P	18.5	24.3	0.00637	0.99945	0.683

**Model**	**Response**	**Parameters**				
		***q***_**e**,**exp**_ (mg g^–1^)	***q***_**e**,cal_ (mg g^–1^)	**k**_**2**_ (g mg^–1^ min^–1^)	*R* ^2^	**RMSE**
pseudo-2nd order kinetic	NH_4_^+^–N	31.4	45.5	0.00026	0.99936	0.438
	NO_3_^–^–N	45.5	103	0.00003	0.99840	1.88
	PO_4_–P	18.5	38.1	0.00011	0.99946	0.733

a Parameters obtained by nonlinear least-squares (GRG, Excel Solver) on qt vs t.

**Table 4:** Modified Langmuir and the Freundlich Isotherm Parameters for the Adsorption of NH_4_^+^–N, NO_3_^–^–N, and PO_4_–P onto TPP-Crosslinked Nano-chitosan under the Investigated Experimental Conditions

Response	Modified Langmuir Isotherm			Freundlich Isotherm		
	*q*_max_ (mg g^–1^)	*K*_ML_	*R* ^2^	*K*_F_	1/n	*R* ^2^
NH_4_^+^–N	50.4 ± 3.14	1680.5 ± 241.1	0.989	3.06 ± 0.577	0.532 ± 0.044	0.979
NO_3_^–^–N	90.2 ± 23.8	1250.2 ± 583.9	0.951	2.16 ± 1.20	0.682 ± 0.129	0.914
PO_4_–P	48.5 ± 12.1	475.1 ± 201.3	0.964	0.964 ± 0.411	0.693 ± 0.097	0.951

For NH_4_^+^–N, the PSO model yielded a lower RMSE than the PFO model, indicating a better empirical fit to the rate profile; however, the PSO *q*_e,cal_ substantially overestimated *q*_e,exp_, whereas the PFO model produced a closer agreement between predicted and experimental equilibrium capacity. This pattern suggests that NH_4_^+^–N adsorption is governed by a combination of mass-transfer and surface interaction mechanisms rather than a strictly second-order surface reaction. For PO_4_–P, the two models produced similar RMSE values and could not be reliably distinguished. For NO_3_^–^–N, both models yielded comparatively high RMSE values and substantial differences between *q*_e,cal_ and *q*_e,exp_, confirming that neither PFO nor PSO adequately captures the adsorption behavior of this species. The Weber–Morris intraparticle diffusion model would provide a more mechanistically appropriate description for NO_3_^–^–N and should be considered in future kinetics analyses.

The results indicate that adsorption is not governed by a single rate-limiting step but rather by a combination of controlling mechanisms, including film mass transfer, intraparticle diffusion, and electrostatic interactions. Notably, direct electrostatic attraction between NH_4_^+^ and the protonated chitosan surface (−NH_3_^+^) is electrostatically unfavorable, as both carry positive charge. NH_4_^+^–N uptake is instead attributed to ionic pairing with negatively charged phosphate domains introduced through TPP crosslinking. These findings demonstrate that model selection must be based on RMSE, *q*_e,cal_, and *q*_e,exp_ agreement in addition to *R* ^2^, as *R* ^2^ alone primarily reflects curve-fitting performance and does not confirm mechanistic validity.

### 3.4 Modified Langmuir and Freundlich Isotherm

The adsorption isotherm parameters obtained for NCS indicate substantial uptake of inorganic nutrients under the tested conditions. Equilibrium data were fitted using the modified Langmuir isotherm proposed by Azizian et al. (2018) for liquid-phase adsorption systems, which yields a dimensionless affinity constant (*K*_ML_) consistent with IUPAC requirements for thermodynamic parameter estimation.^42^ For NH_4_^+^-N, the modified Langmuir model yielded a maximum adsorption capacity (*q*_max_) of 50.4 ± 3.14 mg g^–1^ and a dimensionless modified Langmuir constant (*K*_ML_) of 1680.5 ± 241.1, with a high coefficient of determination (*R* ^2^ = 0.989). The Freundlich model produced a K_F_ value of 3.06 ± 0.577, a 1/n value of 0.532 ± 0.044, and an *R* ^2^ of 0.979.

For NO_3_^–^–N, the modified Langmuir fitting resulted in *q*_max_ = 90.2 ± 23.8 mg g^–1^ and *K*_ML_ = 1250.2 ± 583.9 (*R* ^2^ = 0.951), whereas the Freundlich model yielded *K*_F_ = 2.16 ± 1.20, 1/*n* = 0.682 ± 0.129, and a lower *R* ^2^ of 0.914. Similarly, for PO_4_–P, the modified Langmuir model provided *q*_max_ = 48.5 ± 12.1 mg g^–1^ and *K*_ML_ = 475.1 ± 201.3 (*R* ^2^ = 0.964), while the Freundlich model produced *K*_F_ = 0.964 ± 0.411, 1/n = 0.693 ± 0.097, and *R* ^2^ = 0.951. The saturation concentration for each nutrient was determined from the aqueous solubility of the salt at 25 °C: approximately 80,000 mg L^–1^ for NH_4_^+^–N, 109,000 mg L^–1^ for NO_3_^–^–N, and 52,000 mg L^–1^ for PO_4_–P.^43^ In all three cases, the experimental initial concentration range (*C*_0_ = 30–90 mg L^–1^) was more than 2 orders of magnitude below the corresponding saturation concentration. Because adsorption reduced the dissolved nutrient concentrations, the resulting equilibrium concentrations (*C*_*e*_) were lower than *C*_0_ and therefore also remained substantially below *C*_*s*_, confirming that *C*_*e*_ < *C*_*s*_ throughout the study. This is consistent with the observation by Azizian et al. (2018) that in most experimental adsorption studies, *C*_e_ < *C*_s_. Under such dilute conditions, the modified and classical Langmuir models are expected to yield comparable parameter estimates because the general mathematical form of the modified isotherm closely resembles that of the classical model. Nevertheless, the modified form was adopted to maintain thermodynamic consistency for liquid-phase adsorption and to provide a dimensionless equilibrium constant, *K*_ML_, consistent with IUPAC requirements for thermodynamic calculations.^23,42^

It is noted that the *K*_ML_ standard errors for NO_3_^–^–N and PO_4_–P are comparatively large relative to their respective fitted values, reflecting the limited precision with which the adsorption affinity constant can be resolved from seven equilibrium data points; this is a recognized limitation of isotherm fitting with small datasets and does not affect the reliability of *q*_max_, whose standard errors remain proportionally smaller for all three nutrients. The models’ performance indicates a marginally superior fit of the modified Langmuir isotherm than the Freundlich model, as evidenced by slightly higher *R* ^2^ values for all three nutrients within the tested concentration ranges. It should also be noted that all Freundlich 1/n values were less than unity, suggesting that energetically heterogeneous surfaces were created by the presence of multiple adsorption sites on the NCS, thereby indicating favorable adsorption behavior. The marginally superior modified Langmuir fit, however, suggests that adsorption occurs primarily on finite-capacity surface sites within the concentration range studied, although this is not conclusive given the evidence of surface heterogeneity provided by the Freundlich model.

The apparent coexistence of modified Langmuir-type behavior and surface heterogeneity may be attributed to the presence of multiple chemically distinct functional groups on TPP-crosslinked nano-chitosan.^44,45^ The chitosan backbone contains protonated amino groups (−NH_3_^+^), which can act as important sites for the electrostatic attraction of anionic species, while the phosphate groups introduced by the TPP crosslinker provide additional ion-exchange and complexation sites for cationic species.^45^ The presence of these different active site types supports the observed heterogeneous adsorption behavior, while the finite number of available effective adsorption sites within the tested concentration range is consistent with the capacity-limited behavior described by the modified Langmuir model.

To contextualize the adsorption performance of NCS relative to other chitosan-based materials, Table 5 compares the maximum adsorption capacities *q*_max_ obtained in this study with values reported for NH_4_^+^–N, NO_3_^–^–N, and PO_4_–P adsorption. It should be noted that all literature values were obtained under single-solute conditions using the Langmuir isotherm, whereas the present study additionally reports multi-nutrient capacities obtained under simultaneous competitive adsorption via RSM optimization.

**Table 5:** Comparison of Maximum Adsorption Capacities *Q*_max_ for NH_4_^+^–N, NO_3_^–^–N, and PO_4_–P by Chitosan-Based Adsorbents

Adsorbent	Nutrient	***q***_**max**_ (mg g^–1^)	Isotherm model	System type	pH	References
TPP-crosslinked NCS	NH_4_^+^–N	50.4	Modified Langmuir	single-solute	4–5	**this study**
		11.5	RSM-optimized	multi-nutrient		
NaA zeolite/chitosan porous hybrid beads	NH_4_^+^–N	36.97	Langmuir	single-solute	5–6	^46^
MgO-biochar nanocomposite (RMgN500)	NH_4_^+^–N	21.32	Langmuir	single-solute	6.5	^47^
TPP-crosslinked NCS	NO_3_^–^–N	90.2	Modified Langmuir	single-solute	4–5	**this study**
		37.8	RSM-optimized	multi-nutrient		
AEAPTS-functionalized chitosan-coated sand	NO_3_^–^–N	35.0–35.9	Langmuir	single-solute	6	^48^
Quaternized chitosan microspheres@MgFe-LDH	NO_3_^–^–N	16.15	Langmuir	single-solute	—	^49^
TPP-crosslinked NCS	PO_4_–P	48.5	Modified Langmuir	single-solute	4–5	**this study**
		5.23	RSM-optimized	multi-nutrient		
Zr (IV)-loaded chitosan-modified cork biochar (CS–CBC–Zr)	PO_4_–P	33.89	Sips (Langmuir–Freundlich)	single-solute	4	^50^
Lanthanum-chitosan-polyacrylamide hydrogel	PO_4_–P	91.16	Langmuir	single-solute	5	^51^

### 3.5 Comparison of Multi-nutrient RSM and Single-Solute Adsorption Performance

It should be noted that the adsorption behavior observed under the RSM experimental conditions reflects a competitive multi-solute system, in which NH_4_^+^–N, NO_3_^–^–N, and PO_4_–P coexist and compete for available functional sites on the NCS surface. This competitive environment differs fundamentally from the single-solute conditions employed in the kinetics and isotherm studies, where the absence of co-existing ions allows the adsorbent to approach its maximum theoretical uptake capacity.

For ammonium, adsorption on TPP-crosslinked nano-chitosan involves a complex interaction pathway. While protonated chitosan amino groups (−NH_3_^+^) exhibit limited direct electrostatic affinity toward the NH_4_^+^ cation, the phosphate groups introduced through TPP crosslinking provide localized negatively charged domains that facilitate ammonium uptake via ionic pairing or secondary electrostatic interactions. Under multi-nutrient RSM conditions, however, externally added PO_4_–P acts as a multivalent and strongly interacting anion, preferentially occupying high-affinity sites associated with protonated amino groups and TPP domains.

This competitive site occupation reduces the effective availability of binding sites for both NH_4_^+^–N and NO_3_^–^–N and disrupts secondary ammonium–phosphate associations that may otherwise enhance NH_4_^+^ uptake in single-solute systems. Consequently, the lower adsorption capacities observed under RSM conditions relative to single–component isotherm experiments are attributed to competitive adsorption effects inherent to multi-nutrient systems, rather than to a reduction in intrinsic adsorbent performance. Similar competitive suppression phenomena have been widely reported for chitosan-based adsorbents in multi-component aqueous environments, particularly where strongly binding multivalent anions are present.

### 3.6 Competitive Adsorption Behavior in Multi-nutrient Systems

A clear reduction in adsorption capacity was observed for all nutrients when they coexisted in solution, indicating that adsorption was limited by competition for a finite number of functional sites and by co-ion interference. The ratio qmultiqsingle reflects the effective availability of adsorption sites in the presence of competing ions, such that a higher suppression value indicates greater competition for these sites and, therefore, a greater reduction in effective site availability.^52,53^ The *q*_multi_ represents the effective operational capacity under multi-nutrient conditions, while the difference between *q*_single_ and *q*_multi_ therefore represents the quantification of the capacity reduction attributable to competition (Table 6), which the capacity remaining after site occupation and/or blocking by coexisting species, a behavior commonly reported in multicomponent adsorption systems.^53,54^ The calculated suppression value therefore represents the fractional reduction in adsorption capacity arising from competitive adsorption effects and should be regarded as a phenomenological indicator of multicomponent adsorption behavior rather than a thermodynamic index of selectivity.

**Table 6:** Single-Solute Capacity, Mixed-Nutrient Capacity, and Suppression Index for NH_4_^+^–N, NO_3_^–^–N, and PO_4_–P

RPesponse	*q*_single_ (mg g^–1^)	*q*_multi_ (mg g^–1^)	Suppression index
NH_4_^+^–N	50.4	11.5	0.772
NO_3_^–^–N	90.2	37.8	0.581
PO_4_–P	48.5	5.23	0.892

NO_3_^–^–N showed the lowest suppression (0.581), retaining a substantial fraction of its single-solute capacity. This behavior suggests that nitrate adsorption occurs primarily through relatively weak, non-specific electrostatic interactions distributed across a broader population of accessible surface regions. Because these interaction sites are not restricted to a small number of high-affinity binding locations, nitrate adsorption remains partially preserved even under competitive conditions, consistent with reports that nitrate is often less strongly affected by competing oxyanions in mixed systems.^52^

In contrast, NH_4_^+^–N exhibited a larger reduction (0.772), indicating greater dependence on more limited interaction domains. Under coexistence conditions, occupation or electrostatic screening of these domains by competing ions reduces the availability of adsorption pathways, thereby lowering their effective adsorption capacity, a typical feature of multicomponent adsorption when several solutes target overlapping functional groups.^54^

PO_4_–P experienced the most pronounced decrease (0.892), with its adsorption capacity strongly reduced under coexistence. This behavior indicates that phosphate relies primarily on a limited population of high-affinity sites, which are rapidly saturated or rendered inaccessible when other ions are present. Competitive studies have similarly shown that phosphate strongly competes for positively charged binding sites and can substantially alter adsorption behavior in mixed-ion environments.^55^

The apparent capacity-reduction order (PO_4_–P > NH_4_^+^ > NO_3_^–^) therefore suggests that adsorption performance in the mixed system is governed primarily by competitive site occupation rather than intrinsic single-solute capacity. Consequently, capacities derived from single-solute experiments represent an upper-bound performance, whereas mixed-system capacities better reflect the operational adsorption capacity expected under realistic, co-ion-containing water conditions.^53^

It should be noted that the salinity effects were not included in this study. However, real wet-market wastewater may contain dissolved salts that increase the ionic strength of the solution. Increasing ionic strength modifies the electrical double layer and produces electrostatic shielding, which weakens the attraction between dissolved ions and charged surface functional groups.^56,57^ Elevated concentrations of background ions, such as Na^+^, may therefore reduce adsorption capacity by weakening electrostatic interactions and partially occupying or screening adsorption sites.^56^ The magnitude of this effect depends on the dominant adsorption mechanism, as adsorption governed by outer-sphere electrostatic interactions is generally more sensitive to ionic strength than adsorption governed by inner-sphere surface complexation.

### 3.7 Potential Implementation and Study Limitations

Although nitrate removal was effective at an initial concentration of 30 mg L^–1^, ammonium and phosphate removal remained insufficient to meet typical effluent discharge standards, including those applied in Malaysia, which are 10 mg L^–1^ of NH_4_^+^–N for upstream water and 20 mg L^–1^ for NH_4_^+^–N for normal inland waters.^58^ Therefore, the NCS adsorption process is not intended as a stand-alone treatment but rather as an intermediate or supplementary step for reducing nutrient levels. Additional post-treatment processes, such as biological polishing, constructed wetlands, or membrane-based treatment, would be required downstream to ensure regulatory compliance. Accordingly, the present material is not proposed for bulk municipal wastewater treatment but may be considered for further evaluation in localized nutrient-rich, low-volume wastewater streams, such as wet-market wastewater and comparable food-handling or agro-processing streams, where concentrations are elevated, but flow rates are comparatively low. However, its suitability for these applications has not yet been demonstrated under actual wastewater or continuous treatment conditions.

A recent review identifies nitrate, ammonium, and phosphorus species as wastewater nutrients of concern and describes adsorption onto sorbents as a route for nutrient capture and recovery, supporting the premise that adsorption functions as a process step within nutrient management rather than a complete treatment train for discharge compliance.^59^ In addition, recent work explicitly targeting simultaneous removal of ammonium nitrogen and phosphate with functionalized biochar underscores that co-removal is non-trivial and often requires specifically engineered sorbents, again supporting the broader motivation for downstream or integrated treatment beyond a single, unoptimized adsorption step.^60^

From an engineering integration perspective, NCS could potentially serve as a targeted polishing unit for confined wastewater streams before sewer discharge or connection to centralized treatment, rather than as a stand-alone compliance technology, consistent with broader nutrient-recovery frameworks that treat sorption as an add-on unit within wastewater management pathways.^59^ In such localized applications, adsorption could theoretically be operated in a batch or holding-tank configuration, with treatment restricted to a defined side stream rather than the full municipal wastewater flow. Nevertheless, the required NCS dosage, hydraulic configuration, particle separation efficiency, and operational stability would need to be established through a pilot-scale evaluation. Such targeted adsorption may reduce fluctuations in dissolved nutrient loads entering downstream polishing units; nevertheless, this potential process benefit was not evaluated in the present batch study.^59^

Regeneration and reuse of nutrient-loaded sorbents have been investigated in other adsorption systems using controllable desorption triggers, including pH adjustment, for reversible nutrient capture and release.^61^ However, no desorption, regeneration, or repeated adsorption cycle experiments were conducted for the NCS developed in this study. Therefore, the applicability of these regeneration approaches to TPP-crosslinked NCS remains unverified, and no conclusions can presently be drawn regarding nutrient desorption efficiency, recovery of adsorption capacity, or multicycle stability.

Alternatively, nutrient-loaded NCS could be investigated in future studies for beneficial reuse as a soil conditioner or slow-release nutrient carrier.^62,63^ Such an application should not be assumed solely from the nutrient-retention results obtained in this study and would require experimental assessment of nutrient-release behavior, nanoparticle leaching, ecotoxicity, material degradation, and environmental safety. This potential route is broadly consistent with circular-economy concepts involving nutrient-loaded sorbents and biochar-based slow-release fertilizers.^59,64^

Nevertheless, the recoverability of fine NCS particles remains a practical limitation. Although centrifugation was used for particle separation in the laboratory experiments, its applicability and economic feasibility at larger treatment volumes were not evaluated. Particle aggregation, incomplete recovery, and adsorbent mass loss may affect both treatment performance and the feasibility of repeated use. This limitation supports further investigation of NCS in contained or side-stream treatment configurations where controlled solid–liquid separation may be possible, rather than its direct application to high-throughput municipal wastewater treatment.

In this study, the approximate material cost of NCS was estimated using a simplified raw material calculation based on the ionotropic gelation synthesis route by using eq 8. A representative chitosan-to-TPP mass ratio of 3:1 was assumed. Since adsorption-based wastewater treatment does not necessarily require pharmaceutical-grade purity, food- or technical-grade reagents were considered appropriate for the preliminary raw material cost estimation.^65,66^ Using representative bulk pricing assumptions (food/technical-grade chitosan: average 30 USD kg^–1^; technical-grade TPP: average 4 USD kg^–1^), the raw material cost of NCS was estimated according to eq 8.

8 $$Cost\;per\;kg\;NCS=\frac{\left(0.75\;kg\times P_{CS}\right)+\left(0.25\;kg\times P_{TPP}\right)}{1.00\;kg}$$

This yields a base-case material cost of approximately 23.5 USD kg^–1^, the total being dominated by the price of CS rather than the crosslinker. To relate material cost to adsorption performance, a representative adsorption capacity of ∼5–40 mg nutrient g^–1^ was used to obtain a screening-level cost-per-removal estimate. Under the base-case cost (0.024 USD g^–1^ adsorbent), the implied cost is approximately 0.59–4.7 USD per gram of nutrient removed, prior to regeneration or reuse.

This calculation represents only a preliminary raw material screening estimate. It does not include synthesis energy, chemical consumption, washing, drying, centrifugation or other particle-separation processes, labor, adsorbent loss, regeneration chemicals, treatment of nutrient-rich desorption solutions, or final disposal. The estimate should therefore not be interpreted as a complete process level economic assessment. Although this estimate remains higher than that of commodity sorbents such as modified biochar that cost around 5–6 USD kg^–1^ depending on feedstock and process conditions, it is substantially lower than laboratory reagent-based estimates and therefore more representative of potential bulk raw material procurement rather than demonstrated practical production costs.^67^

Overall, the present results provide proof-of-concept evidence for simultaneous nutrient adsorption by NCS under controlled synthetic wastewater conditions. Practical applicability, regeneration, reusability, and economic feasibility remain to be established through actual-wastewater testing, effective particle-recovery methods, and multi-cycle adsorption–desorption experiments that quantify desorption efficiency, adsorption-capacity retention, nanoparticle aggregation, structural changes, and adsorbent mass loss.

## 4 Conclusion

NCS was successfully synthesized via ionotropic gelation and applied to the simultaneous adsorption of NH_4_^+^–N, NO_3_^–^–N, and PO_4_–P from synthetic wastewater representing nutrient-rich wet-market effluent. Characterization confirmed the formation of stable nanoscale particles with a surface charge suitable for ionic interaction with dissolved nutrients, and RSM showed that adsorbent dosage, contact time, and initial concentration significantly governed adsorption performance, with the reduced models providing reliable predictions within the investigated experimental range. Kinetic analysis indicated faster uptake of ammonium and phosphate than nitrate: the pseudo-second-order model gave the best empirical fit for NH_4_^+^–N and a comparable fit for PO_4_–P, but the marked deviation between calculated and measured equilibrium capacities indicates that uptake is governed by a combination of mass-transfer and surface interactions rather than a true second-order reaction, while nitrate followed a diffusion-influenced process that neither model captured adequately and that warrants intraparticle-diffusion (Weber–Morris) analysis in future work.

Although single-solute isotherms yielded high theoretical maximum capacities, these were substantially reduced under multinutrient conditions, where ammonium, nitrate, and phosphate compete for a limited set of interacting functional domains, with suppression following the order phosphate > ammonium > nitrate. Single-solute capacities therefore represent an upper bound rather than the operational performance expected in real wastewater streams. Accordingly, NCS is best positioned for further investigation as a supplementary or polishing adsorbent for localized, nutrient-rich side-stream wastewater treated at moderate flow rates in batch- or tank-type configurations. More broadly, these findings demonstrate that multi-component evaluation provides a more realistic measure of adsorbent performance for practical wastewater treatment than single-solute studies alone.

As actual wastewater contains competing ions, organic matter, suspended solids, and other matrix components, further testing using actual wastewater is required before practical applicability can be established. Regeneration and reusability were not experimentally investigated in this study. Although the original nanoparticle morphology remained visually identifiable after one adsorption experiment, this observation does not demonstrate nutrient desorption, recovery of adsorption capacity, or stability during repeated use. Multi-cycle adsorption–desorption experiments assessing desorption efficiency, capacity retention, nanoparticle aggregation, particle recovery, and adsorbent mass loss are therefore required. The absence of these data represents a key limitation of the present study, and NCS should not yet be considered a demonstrated regenerable or reusable adsorbent.

## Supplementary Material



## Data Availability

The data that support the findings of this study are available from the corresponding author, Norli Ismail, upon reasonable request.

## Notes

Table S1: Full ANOVA results and model fit statistics (F-values, *p*-values, *R* ^2^, adjusted *R* ^2^, predicted *R* ^2^, and adequate precision) for the reduced quadratic polynomial models describing NH_4_^+^–N, NO_3_^–^–N, and PO_4_–P adsorption capacity (PDF)
