# Supplementary material for: Performance of TPP-Crosslinked Nano-chitosan for Simultaneous Nutrient Adsorption in Nutrient-Rich Wastewater
Source: ACS Omega. 2026 Sep 15;11(38):57117–31. doi: 10.1021/acsomega.6c04170 (PMC13629023; doi:10.1021/acsomega.6c04170)
Supplement: Supplementary file 1 [file ao6c04170_si_001.pdf]

Performance of TPP-Crosslinked Nano-chitosan for Simultaneous Nutrient Adsorption in Nutrient-Rich Wastewater

Li Kai Chin, [†] Mamoru Oshiki, [‡] and Norli Ismail[*, †]

[†]Environmental Technology Division, School of Industrial Technology, Universiti Sains Malaysia, Penang 11800, Gelugor, Malaysia

[‡]Division of Environmental Engineering, Faculty of Engineering, Hokkaido University, North-13, West-8, Kita-ku, Sapporo, 060-8628, Japan

*Email: norlii@usm.my.   Phone: +60 46532824.

Table S1 Full ANOVA results and model fit statistics (F-values, p-values, R2, adjusted R2, predicted R2, and adequate precision) for the reduced quadratic polynomial models describing NH4+–N, NO3—N, and PO4–P adsorption capacity

Source	NO_3^-–N		NH_4^+–N		PO_4–P	
	F-value	*p*-value	F-value	*p*-value	F-value	*p*-value
**Model**	47.1	< 0.001	42.0	< 0.001	45.2	< 0.001
A	19.5	0.002	67.5	< 0.001	2.27	0.160
B	40.3	0.001	78.2	< 0.001	120	< 0.001
C	210	< 0.001	16.7	0.002	21.2	0.001
AB	-	-	-	-	-	-
AC	40.6	0.001	-	-	13.3	0.004
BC	3.83	0.082	-	-	-	-
A^2	9.53	0.013	6.21	0.03	-	-
B^2	4.78	0.057	43.3	< 0.001	68.8	< 0.001
C^2	-	-	-	-	-	-
Lack of Fit	2.51	0.196	0.397	0.865	3.88	0.104

Fit Statistics

	NO_3^-–N	NH_4^+–N	PO_4–P
R^2	0.973	0.950	0.954
Adjusted R^2	0.953	0.928	0.933
Predicted R^2	0.859	0.895	0.842
Adeq Precision	27.2	22.9	22.8

A= Initial dosage, B= Initial concentration of pollutant, C = Contact time
